# Supplementary material for: Identification of photoperiod-induced specific miRNAs in the adrenal glands of Sunite sheep (Ovis aries)
Source: Front Vet Sci. 2022 Jul 22;9:888207. doi: 10.3389/fvets.2022.888207 (PMC9354845; doi:10.3389/fvets.2022.888207)
Supplement: Supplementary file 1 [file Data_Sheet_1.ZIP › Supplementary file/miRDeep2 file.zip!/miRDeep2 file/Know_Structure/LP42A3/oar-mir-152.pdf]

miRBase precursor : oar-mir-152
Total read count : 13316
oar-miR-152 read count : 13316
remaining reads : 0

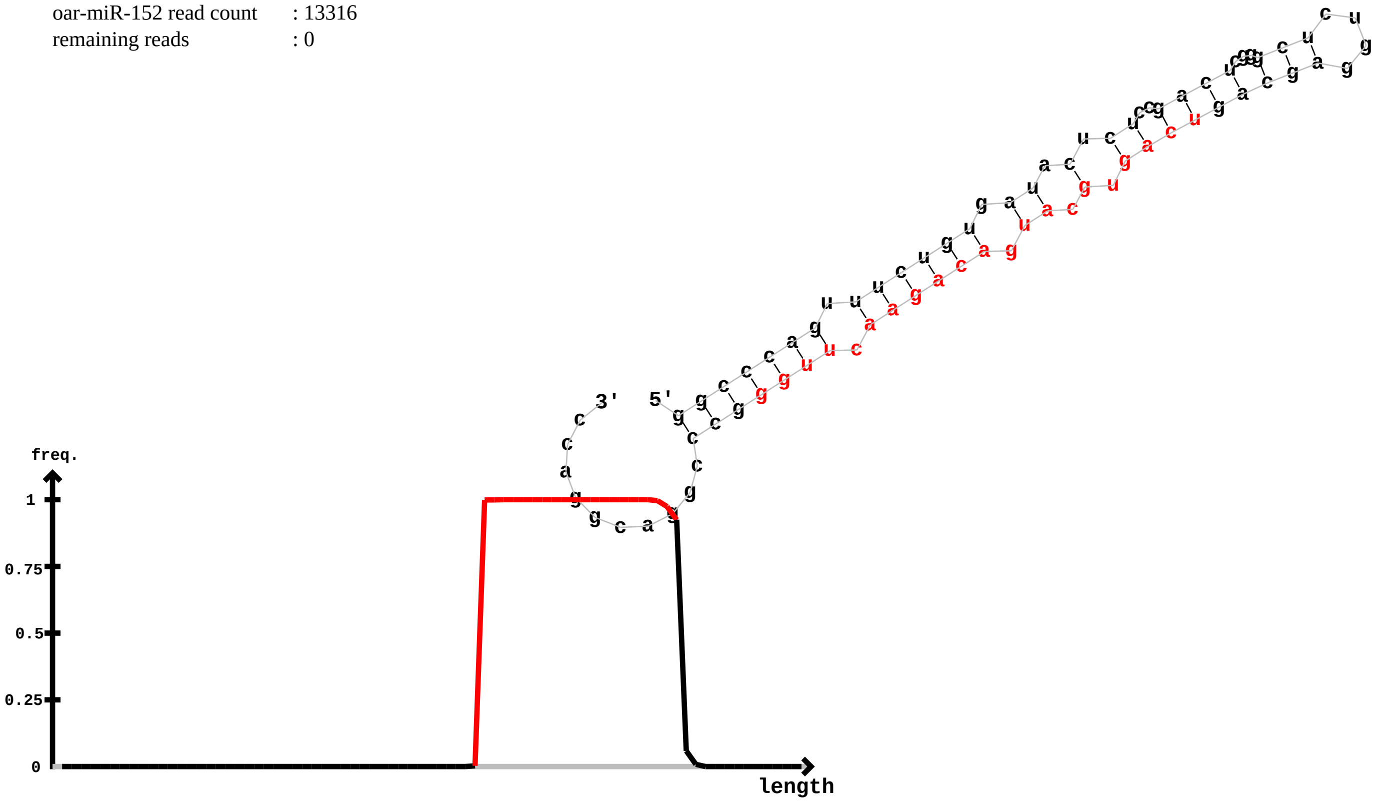


```
                                            oar-miR-152
5'- ggcccaguuucugugauacucuccgacucgggcucuggagcagucagugcaugacagaacuugggcccggacggacc   -3'    exp
    (((((((.((((((.((.(.((..((((...(((....))))))))).).)).)))))).)))))))..........     reads      mm         sample
    ..........................................gucagugcaugacagaacuu...............     5          0          seq
    ..........................................gucagugcaugacagaacuug..............     4          0          seq
    ..........................................gucagugcaugacagaacuugg.............     25         0          seq
    ...........................................ucagugcauCacagaacu................     8          1          seq
    ...........................................ucagugcaugacUgaacu................     1          1          seq
    ...........................................ucagugcaugacagaacu................     38         0          seq
    ...........................................ucagugcauCacagaacuu...............     17         1          seq
    ...........................................ucagugcaugacagaacuu...............     275        0          seq
    ...........................................ucagugcaugacagaacuug..............     651        0          seq
    ...........................................ucagugAaugacagaacuug..............     2          1          seq
    ...........................................ucagugcaugacagaacuugg.............     11495      0          seq
    ...........................................ucagugcaugacagaacuuggg............     672        0          seq
    ...........................................ucagugcaugacagaacuugggc...........     110        0          seq
    ............................................cagugcaugacagaacuugg.............     3          0          seq
    .............................................Ugugcaugacagaacuugg.............     1          1          seq
    .............................................agugcaugacagaacuugA.............     2          1          seq
    .............................................agugcaugacagaacuugg.............     7          0          seq
```
